# Mixture design approach for the development of reduced fat lamb patties with carboxymethyl cellulose and inulin

**DOI:** 10.1002/fsn3.965

**Published:** 2019-02-19

**Authors:** Juliana M. Guedes‐Oliveira, Bruno R. C. Costa‐Lima, Denize Oliveira, Adelino Neto, Rosires Deliza, Carlos A. Conte‐Junior, Carlos Frederico M. Guimarães

**Affiliations:** ^1^ Faculdade de Veterinária, Departamento de Tecnologia de Alimentos Universidade Federal Fluminense Niterói Brazil; ^2^ Departamento de Tecnologia de Alimentos Instituto Federal de Educação, Ciência e Tecnologia da Paraíba Sousa Brazil; ^3^ Department of Animal and Food Sciences University of Kentucky Lexington Kentucky; ^4^ Embrapa Agroindústria de Alimentos Rio de Janeiro Brazil; ^5^ Instituto de Química, Centro de Tecnologia Universidade Federal do Rio de Janeiro Rio de Janeiro Brazil

**Keywords:** carboxymethyl cellulose, fat replacer, inulin, lamb patties, mixture design

## Abstract

Fat replacement by carboxymethyl cellulose (CMC) and inulin (IN) for the manufacture of low‐fat lamb patties was investigated utilizing mixture design. The effect of fat, CMC, and IN levels on texture, color, weight loss, patty diameter reduction, and sensory characteristics was investigated. The presence of CMC decreased hardness (*p* < 0.05). While CMC and IN also decreased springiness, cohesiveness, gumminess, and chewiness (*p* < 0.05), no effect on adhesiveness was observed (*p* > 0.05). CMC increased L* (lightness), a* (redness), and b* (yellowness) values in raw patties, whereas IN and fat contributed to a decrease on these parameters. Higher contents of CMC resulted in products with lower weight loss (*p* < 0.05) with no significative diameter reduction (*p* > 0.05). Nonetheless, higher levels of CMC affected the sensory acceptance resulting on products described as crumbly and with residual flavor by check‐all‐that‐apply questions. CMC and IN can be used as fat replacers in lamb patties; however, the content of each ingredient must be carefully considered. In this study, it was observed that contents of CMC higher than 1% (w/w) negatively affected the product, whereas IN levels were not capable to decrease weight loss and diameter reduction in lamb patties.

## 1 INTRODUCTION

In the last few decades, the food industry has shifted its focus toward mitigating some negatively perceived traits in processed foods in order to positively influence the consumer purchase (Shan et al., 2017). Meat products are rich sources of proteins, vitamins, and minerals; however, the high contents of saturated fatty acids, cholesterol, and salt (Jiménez‐Colmenero & Cofrades, 2001) negatively affect the consumer perception by associating meat products with an unhealthy diet (Hygreeva, Pandey, & Radhakrishna, 2014). Because fat is an important ingredient in processed meats, the decrease on the fat content usually negatively affects final product appearance, flavor, and texture (Furlán, Padilha, & Campderrós, 2014; Piñero et al., 2008). Recent studies have shown that some ingredients can be added as fat replacers in meat products, such as inulin and cellulose fibers, as microcrystalline cellulose (MCC) or carboxymethyl cellulose (CMC) (Álvarez & Barbut, 2013; Furlán et al., 2014; Gibis, Schuh, Allard, & Weiss, 2017; Gibis, Schuh, & Weiss, 2015; Mittal & Barbut, 1993; Schuh et al., 2013; Tomaschunas et al., 2013).

Carboxymethyl cellulose and inulin are of growing interest by the muscle food industry especially because of health‐related characteristics in addition to the meat binding capabilities (Arancibia, Costell, & Bayarri, 2011). The technological importance of CMC and inulin involves the increase on the water‐holding capacity and gelling characteristics (Gibis et al., 2015; Tomaschunas et al., 2013). CMC is obtained from cellulose after heating with alkali and posterior reaction with chloroacetic acid, leading to the etherification of the hydroxyl groups with methylcarboxyl groups (Gibis et al., 2015; Schuh et al., 2013). Inulin is a fructan varying in length from 2 to 60 fructose units linked by β‐(2 → 1) glycosidic bonds (Lopes et al., 2017; Luo et al., 2017). In addition, inulin is a soluble dietary fiber considered a functional ingredient (Álvarez & Barbut, 2013), and its prebiotic effect is intensified when combined with CMC (Juśkiewicz & Zduńczyk, 2004).

Patties are an attractive subject for fat reduction strategies due to the usual high fat content and popularity (Selani et al., 2016). This meat product is convenient and is widely consumed despite of their potential negative impact on consumer's health (Rodríguez‐Carpena, Morcuende, & Estévez, 2012). Brazilian consumers consider lamb meat healthy and nutritive; however, its consumption is associated with special occasions and varies between the country's regions; when asked about the low consumption of lamb meat products, most of consumers attribute it to the lack of habit and limited availability (Andrade, Sobral, Ares, & Deliza, 2016). In addition, lamb burgers are a viable alternative to add value to lamb meat processing chain (Fernandes et al., 2017).

Mixture design methodology can be used to study the ingredient functionality in processed foods, and the systematic experimental design validates the importance of ingredient interactions (García‐García & Totosaus, 2008; Marchetti, Argel, Andrés, & Califano, 2015). Mixture design is a specialized form of response surface methodology (RSM), where the sum of all the components must be 1 or 100%; the components of a mixture cannot be varied independently; and the object of the study is not the effect of the variation of the absolute quantity of the variables, but the effect of the variation of the ratios among the variables (Keenan, Resconi, Kerry, & Hamill, 2014; Leardi, 2009). Applying this methodology, the objective of this work was to investigate the effect of replacing fat by different combinations of carboxymethyl cellulose and inulin on color, texture properties, and sensory quality of lamb patties.

## 2 MATERIALS AND METHODS

### 2.1 Experimental design

**Figure 1:**
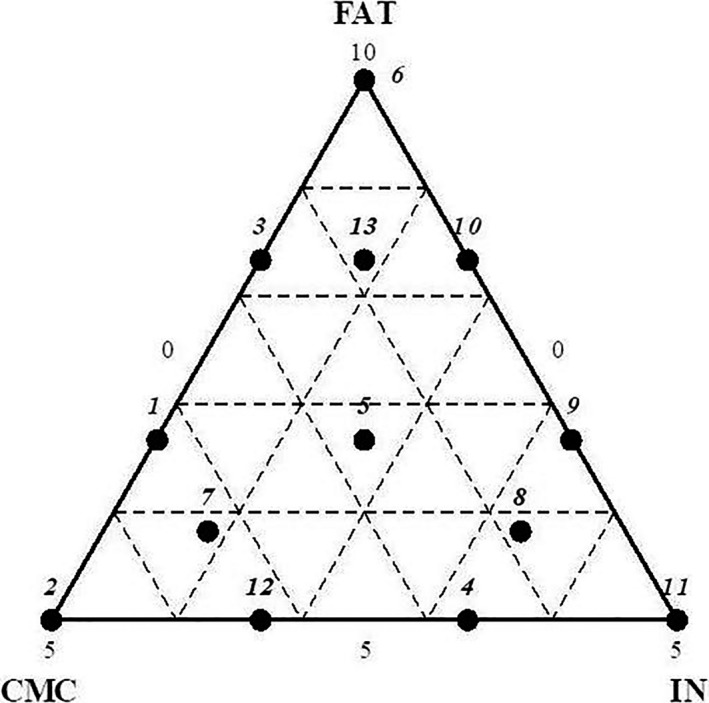
Thirteen points simplex lattice mixture design for the effects of fat (X_1_), Carboxymethylcellulose (X_2_), and inulin (X3) in lamb patties

In the present study, simplex lattice mixture design (SLMD), which included 13 combinations (Table 1) of three ingredients, namely fat (X_1_, FAT), carboxymethylcellulose (X_2_, CMC), and inulin (X_3_, IN), was performed. The sum of FAT, CMC, and IN was set at 10% (w/w), and the minimum percentage of fat was set at 5% (w/w; Figure 1). The centroid point formulation (5) was prepared three times. The goal of the mixture design is to find the best mixture of the three ligands (X_1_, FAT; X_2_, CMC; X_3_, IN) to develop the best product in terms of texture, color, and sensory acceptance. The equation of the following model with three‐ingredient interaction term was:

(1) $$Y=b_{1}X_{1}+b_{2}X_{2}+b_{3}X_{3}+b_{12}X_{1}X_{2}+b_{13}X_{1}X_{3}+b_{23}X_{2}X_{3}+b_{123}X_{1}X_{2}X_{3}\text{,}$$

where:

**Table 1:** Experimental design of three components in lamb patties formulation

Mixtures	Ingredient proportions		
	X_1_	X_2_	X_3_
1	6.7	3.3	0.0
2	5.0	5.0	0.0
3	8.3	1.7	0.0
4	5.0	1.7	3.3
5	6.6	1.7	1.7
6	10.0	0.0	0.0
7	5.9	3.3	0.8
8	5.9	0.8	3.3
9	6.7	0.0	3.3
10	8.3	0.0	1.7
11	5.0	0.0	5.0
12	5.0	3.3	1.7
13	8.4	0.8	0.8

X_1_: fat; X_2_: carboxymethylcellulose; X_3_: inulin.

X_1_ + X_2_ + X_3_ = 10% (w/w).

*Y*—is a predictive dependent variable,

*b*_1_,* b*_2_,* b*_3_, *b*_12_,* b*_13_,* b*_23_, and *b*_123_ *—*predictors of the regression coefficients for X_1_, X_2_, and X_3_.

X_1_, X_2_, and X_3_—the interaction terms.

### 2.2 Lamb patties formulation and manufacture

A total of 50 lamb top sirloin (IMPS# 234G; USDA, 2014) from the left side of the carcasses were procured from Cordeiro Rei (Americana, São Paulo, Brazil). Fat and visible connective tissue were manually trimmed, and the lean meat was ground using a 6‐mm plate (B5509; Botini, São Paulo, Brazil). The ground lamb meat (87.5%) was combined with garlic powder (0.3%), onion powder (0.3%), sodium chloride (1.5%), monosodium glutamate (0.3%), ground pepper (0.1%), vegetable fat (hydrogenated soybean oil), CMC, and IN as illustrated in Table 1. Hydrogenated soybean oil was used instead of the trimmed fat because the top sirloin contained insufficient content of fat to reach the quantity needed to each mixture. After manual homogenization for three minutes, the batches of 1 kg of each of the 13 mixtures were manually formed into patties of 59 mm diameter and weighing 30 g. Patties were placed over soaker pads on Styrofoam trays, overwrapped with oxygen permeable PVC film, and stored at 4°C for 24 hr until analyses.

### 2.3 Instrumental color analysis

Surface color of raw lamb patties was measured using a portable spectrophotometer (CM‐600D; Konica Minolta Sensing Inc., Osaka, Japan) equipped with illuminant D65, 8 mm aperture, and 10° standard observer. Lightness (L* value), redness (a* value), and yellowness (b* value) parameters were recorded from two random spots from ten patties per mixture following standard procedures (AMSA, 2012).

### 2.4 Instrumental texture analysis, weight loss, and diameter reduction

Ten patties for each formulation (Table 1) were cooked to 75°C of internal temperature using an air fryer (Model: HD9240, Philips, Brazil). The cooked patties were subjected to Texture Profile Analysis using a TX. XT plus texture analyzer (Stable Micro System, London, UK) equipped with a 75‐mm diameter cylindrical metal probe. Texture profile analysis was performed according to the method of Bourne (1978) described by Claus and Sørheim (2006). Patties were compressed to 70% of their initial weight in two cycles at pretest speed of 5 mm/s, test speed of 1 mm/s, and post‐test speed of 5 mm/s. The time between compressions was 2 s. The evaluated parameters were hardness (N), adhesiveness (g·s), chewiness (g·cm), gumminess (dimensionless), springiness (mm), and cohesiveness (dimensionless).

Weight loss was calculated considering the initial (raw sample) and final (cooked sample) weights, and expressed as percentage of the initial weight as follows: Weight loss (%) = (final weight)/(initial weight) ×100. Diameter reduction was calculated according to the following equation: diameter reduction (%) = (raw diameter − cooked diameter)/(raw diameter) ×100 (Heck et al., 2017).

### 2.5 Sensory analysis and caloric value

A preliminary screening with the 13 patties was carried out, taking into account the sensory attributes of the patties; therefore, the formulations with more than 1.7% (w/w) of CMC were rejected because of the extremely soft texture and residual flavor. Furthermore, the acceptability of patties is positively correlated with appearance, texture, and flavor; thus, those mixtures not rejected at the preliminary screening and similar in terms of yield and texture parameters were selected, and their sensory characteristics were identified in a preliminary test by ten consumers randomly recruited at Embrapa Food Technology (Rio de Janeiro, RJ, Brazil). Cooked patties (see item 2.4) were halved, kept at 55°C, and presented to the 10 participants for the elicitation of the descriptive terms of appearance, aroma, texture, flavor, and residual flavor. The most mentioned terms were chosen to compose the check‐all‐that‐apply (CATA) questions and can be seen in Table 3. CATA questions consist of a list of words or phrases from which respondents should select all the words they consider appropriate to describe a product (Ares, Deliza, Barreiro, Giménez, & Gámbaro, 2010). One hundred consumers participated in the test were recruited within the staff and interns of Embrapa Food Technology. The participants received the sample in the semi conditions described previously and were asked to try the samples and to indicate their overall liking using a 9‐point hedonic scale (1 = dislike very much, 9 = like very much) and to answer a CATA question composed of eighteen sensory terms (Table 3), selected based on results from preliminary studies. The order of the terms was balanced across participants, and the samples were presented one by one, following a Williams’ Latin square design. Data were collected on laptops using software Fizz version 2.14 (Biosystemes, Courtenon, France).

Those mixtures selected had the caloric values calculated according to ANVISA (2005). The caloric value of cooked patties was calculated using the formula: Calories= (C ∗ 4) + (P ∗ 4) + (F ∗ 9). C = Carbohydrate content (g/100 g), P =  Protein content (g/100 g), F = Fat content (g/100 g).

### 2.6 Statistical analyses of data

Mixture design, generation of responses surfaces, and contour plot were accomplished using Minitab version 17 Software (Minitab Inc., State College, PA, USA). Analysis of variance (ANOVA) was performed on consumers overall liking scores and caloric value. Differences were estimated using Tukey's test and were considered significant when *p* < 0.05. Cluster analysis was used to segment consumers with similar preferences by hierarchical analysis using Euclidean distances and the Ward method to group individuals. Cochran's *Q* test was used to evaluate differences among samples for each term of the CATA questions.

## 3 RESULTS AND DISCUSSION

### 3.1 Texture analysis

**Figure 2:**
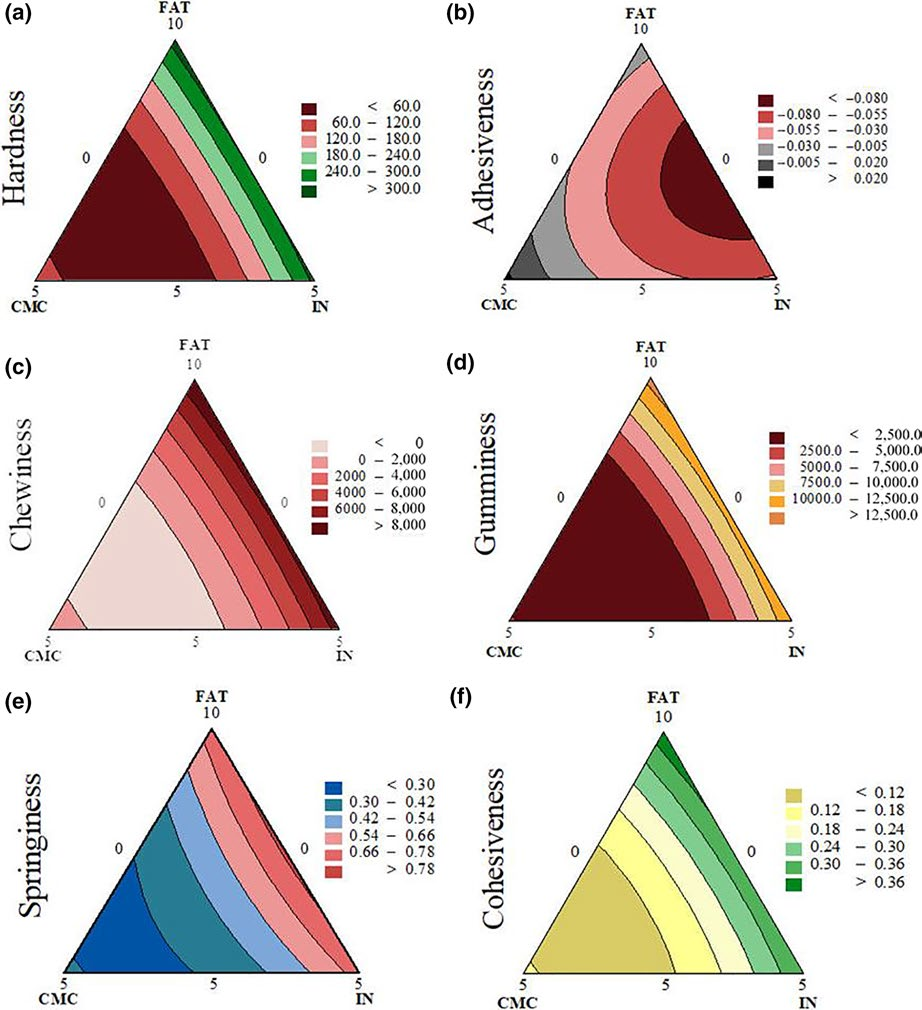
Contour plots of (a) hardness (N); (b) adhesiveness (g·s); (c) chewiness (g·cm); (d) gumminess; (e) springiness (mm); and (f) cohesiveness

**Figure 3:**
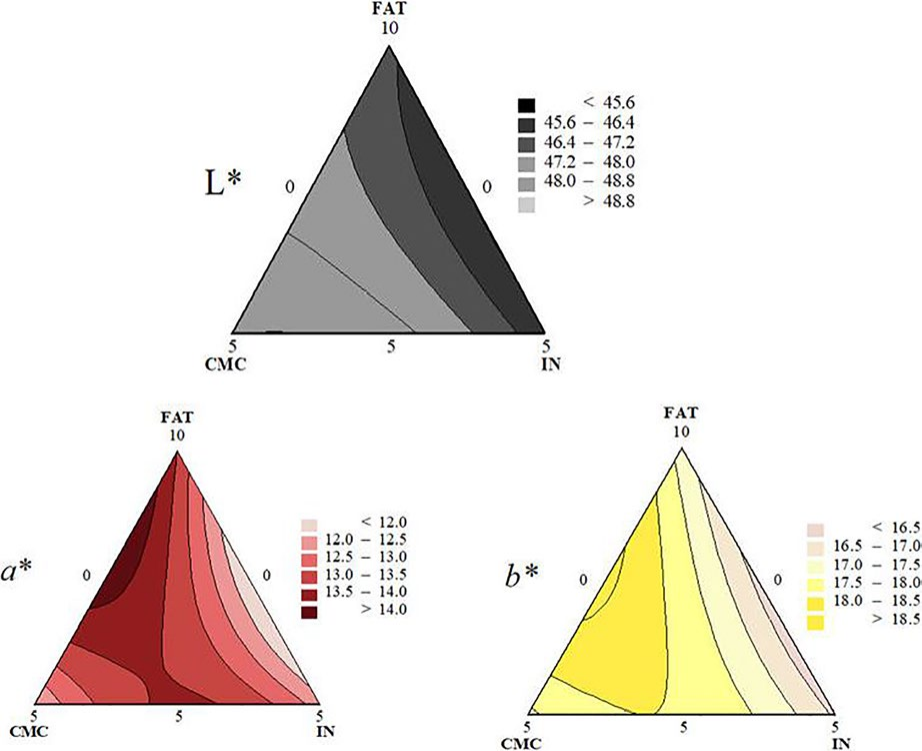
Contour plots of color parameters *L* (lightness); a* (redness); and b* (yellowness) values

**Figure 4:**
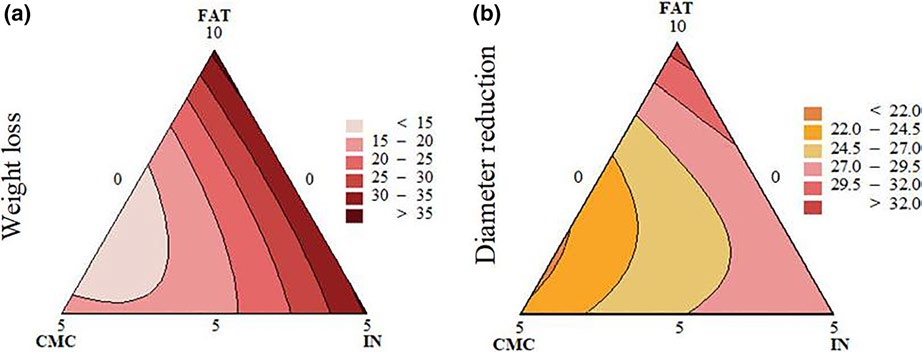
Contour plots of (a) weight loss (g/100 g); (b) diameter reduction (%)

Table 2 presents the regression coefficients and significance level (*p*‐value) of texture parameters of the evaluated lamb patties. Figures 2, 3, 4 represent the predicted values of the response surface models as contour plots. In the present study, hardness, springiness, cohesiveness, gumminess, and chewiness were affected by interactions between the three ingredients (*p* < 0.05; Table 2; Figure 2).

**Table 2:** Regression coefficient and *p* value (in parenthesis) of the instrumental texture and color parameters, diameter reduction, and weight loss of lamb patties formulations

	FAT	CMC	IN	FAT × CMC	FAT × IN	CMC × IN
Hardness	327 (n/a)	1,534 (n/a)	462 (n/a)	−3,269 (**0.010**)	−320 (0.740)	−3,219 (**0.01**0)
Adhesiveness	−14 (n/a)	361 (n/a)	414 (n/a)	−592 (0.733)	−1,012 (0.563)	−520 (0.764)
Chewiness	9,882 (n/a)	49,967 (n/a)	15,208 (n/a)	−111,329 (**0.014**)	−14,623 (0.681)	−103,794 (**0.019**)
Gumminess	13,764 (n/a)	66,913 (n/a)	25.537 (n/a)	−149,059 (**0.014**)	−30,107 (0.532)	−135,957 (**0.021**)
Springiness	0.723 (n/a)	1.640 (n/a)	0.042 (n/a)	−3.425 (**0.017**)	1.328 (0.269)	−3.199 (**0.023**)
Cohesiveness	0.405 (n/a)	1.158 (n/a)	0.594 (n/a)	−2.580 (**0.010**)	−0.561 (0.469)	−2.216 (**0.019**)
L*	46.546 (n/a)	50.703 (n/a)	49.432 (n/a)	0.478 (0.990)	−8.708 (0.824)	16.196 (0.680)
a*	13.49 (n/a)	−2.04 (n/a)	21.42 (n/a)	24.94 (0.306)	−20.80 (0.388)	23.63 (0.330)
b*	17.289 (n/a)	7.286 (n/a)	19.371 (n/a)	20.339 (0.107)	−7.642 (0.510)	16.814 (0.170)
Diameter reduction	33.31 (n/a)	46.81 (n/a)	41.58 (n/a)	−70.78 (0.204)	−35.25 (0.508)	21.14 (0.688)
Weight loss	36.40 (n/a)	103.9 (n/a)	47.6 (n/a)	−212.0 (**0.035**)	−25.8 (0.760)	−127.0 (0.161)

Bold values are *p* < 0.05.

n/a means not applicable because in the mixture design the linear coefficient of each factor is always considered.

Hardness ranged from 45.2 to 342.1 N and was dependent on CMC, FAT, and IN contents. The highest regression coefficient corresponds to CMC reflecting its greater contribution to this attribute than FAT and IN. One can observe on Figure 2a that greater levels of CMC negatively affected hardness values. Gibis et al. (2015) reported that the inclusion levels of CMC more than 2% (w/w) weakened the protein network between the fat and meat particles. Other authors also reported a negative effect on high levels of CMC on gel strength of meat products (Gibis et al., 2017; Schuh et al., 2013). For inulin, Keenan et al. (2014) reported greater hardness values due to the increase on the inulin content in sausages. In contrast, Han and Bertram (2017) demonstrated that hardness of a model meat product with inulin exhibited no difference when compared to the control counterpart. Furthermore, Garcia, Cáceres, and Selgas (2006) concluded that inulin powder tends to increase the hardness of meat products, whereas inulin gel produces softer products. Han and Bertram (2017) attributes the increase in hardness to the ability of dietary fiber to promote and strengthen connections between the various matrix components. Also, the differences in textural properties depend on the characteristics of dietary fibers, as molecular weight and hydrophobicity that cause differences in their water solubility, viscosity enhancement, opacity, surface activity, and binding capacity.

Adhesiveness was not affected (*p* > 0.05) by the interactions between FAT*CMC, FAT*IN or CMC*IN. Nonetheless, the individual contribution of IN was greater than CMC, and FAT level negatively contributed to the adhesiveness value (Table 2). CMC was the major (highest linear coefficient) contributing factor to springiness, cohesiveness, gumminess, and chewiness. All these parameters were affected (*p* < 0.05) by the interaction coefficients between FAT and CMC, and CMC and IN. CMC*IN contributed to the decrease on springiness, cohesiveness, gumminess, and chewiness values. According to Andrès, Zaritzky, and Califano (2006), chewiness is directly related to hardness, cohesiveness, and springiness; thus, a decrease on these parameters potentially results on decreased chewiness. Han and Bertram (2017) reported that CMC decreased the hardness and chewiness of a model meat product, whereas the product containing inulin was not different when compared to the control counterpart.

### 3.2 Instrumental color

The instrumental color was conducted on the surface of raw lamb patties. The raw product color was chosen as this parameter drives consumer purchase decision of muscle foods and discoloration is perceived as unwholesomeness (Carpenter, Cornforth, & Whittier, 2001). Nonetheless, the majority of previous studies dealing with CMC and inulin as fat replacement ingredients utilized cooked sausages.

The evaluated color parameters were affected solely by the linear coefficients of FAT, CMC, and IN but not their interactions (Table 2 and Figure 3). Figure 3 shows that mixture without CMC presented lower lightness. Álvarez and Barbut (2013) also observed a decrease in L* when inulin was added to cooked meat batters and Felisberto, Galvão, Picone, Cunha, and Pollonio (2015) when inulin was added to raw beef batters. Keenan et al. (2014) also reported that the inulin inclusion contributed to a decrease on lightness and yellowness on pork sausage. In contrast, the addition of inulin tended to increase the lightness of chicken and pork‐beef sausage, reaching values similar to the control with more fat content (Mendoza, Garcia, Casas, & Selgas, 2001; Menegas, Pimentel, Garcia, & Prudencio, 2013). Angiolillo, Conte, and Del Nobile (2015) explain that fructooligosaccharides (FOS) and inulin can interact with the meat protein component creating a sort of gel that coexists with the other meat components yielding a linked and consistent unit that contributes to the loss of lightness.

Mixtures with low CMC and high FAT and IN presented the lowest redness, whereas mixture with high FAT and levels of CMC presented highest redness (Figure 3 and Table 1). Mittal and Barbut (1993) documented an increase on raw pork sausage redness when added with CMC. The redness decreases significantly with an increase in fat content mainly because of lower exposure of lean meat content (Crehan, Hughes, Troy, & Buckley, 2000).

Mixtures with high FAT and IN presented the lowest yellowness. Keenan et al. (2014) also observed that inulin inclusion contributed to a decrease on yellowness on pork sausage. Felisberto et al. (2015) observed no differences in yellowness on raw meat batters containing 3% and 6% of inulin. Although yellowness corresponds to concentrations of fat in the product (Schuh et al., 2013), inulin forms white translucent gels with no dominant color (Cáceres, García, Toro, & Selgas, 2004). Gibis et al., (2015) observed that CMC levels of 1%, 2%, and 3% promoted an increase on yellowness and lightness on grilled beef patties.

### 3.3 Weight loss and diameter reduction

Carboxymethyl cellulose and IN levels exhibited greater contribution (Table 2) to diameter reduction and weight loss parameters than FAT level. Furthermore, weight loss was only affected by the interaction between FAT and CMC (*p* < 0.05; Table 2; Figure 4). Greater CMC content in the mixture contributed to lower weight loss and smaller diameter reduction. In addition, the IN level was not capable of mitigating neither weight loss nor diameter reduction in mixtures without or with low levels of CMC, potentially due to the role of CMC on moisture retention (Gibis & Weiss, 2017), or due to a less dense meat protein matrix because of high fat level (Piñero et al., 2008). The presence of inulin makes the gel structure more compact during cooking preventing proteins from holding water, resulting on increased weight loss (Cáceres et al., 2004; Carballo, Fernandez, Barreto, Solas, & Colmenero, 1999). In addition, the ability of a meat system to hold water is dependent of the strength of the protein network developed and the capacity of the hydrocolloid to entrap water within it, and the CMC is an anionic water‐soluble polymer, which likely interacts with meat proteins (Han & Bertram, 2017). Nonetheless, Álvarez and Barbut (2013) observed a decrease on the cooking loss when inulin was incorporated in meat batters. The CMC also contributed to mitigate the patty diameter shrinking due to cooking (Figure 4). This result agrees with the observations of Piñero et al. (2008) who reported that the size and shape of low‐fat beef patties were less affected by cooking due to the binding and stabilizing property added oat soluble fiber.

### 3.4 Sensory analysis and calorific value

Samples M3, M4, M5, and M8 were selected for sensory evaluation, and the results of the acceptance evaluation and the CATA questions are presented in Table 3. Based on the overall liking, the consumers rated the M8 higher acceptance (*p* < 0.05) than the mixtures 3, 4, and 5. The lamb patties M8 had higher frequency of mention for the terms “firm texture,” “homogeneous,” “salty,” “meat aroma,” “seasoned,” “tasty,” “pleasant aroma,” “little salty,” “little seasoning,” “lamb flavor,” “juicy,” and “barbecue aroma than M3, M4, and M5.” The terms with more frequency of use for M3, M4, and M5 were “fatty,” “tender texture,” “residual flavor,” “crumbly,” “residual fat,” and “moisty appearance.” Taking into account the results of the acceptance evaluation and the sensory description of samples, it is possible to suggest that the characteristics “Fatty,” “tender texture,” “residual flavor,” “crumbly,” “residual fat,” and “moist appearance” had a negative impact on product perception. Table 3 shows the terms used by participants to describe lamb patties and the number of mentions. The terms that were related with the lower acceptance of mixtures 3, 4, and 5 as “residual flavor”; “moisty appearance”; “fatty”; “tender texture”; “residual fat”; and “crumbly” were much less mentioned when assessing the M8 sample.

**Table 3:** Overall liking and terms used by consumers for describing the lamb patties and number of mentions

	Samples			
	Mixture 3	Mixture 4	Mixture 5	Mixture 8
Overall liking^a^	6.4^a^±0.4ral	6.3^a^ ± 1.86	6.2^a^ ± 1.78	7.8^b^± 1.18
Moisty appearance*	40	50	60	25
Homogeneous*	14	12	11	32
Fatty*	54	53	57	16
Juicy	42	43	40	51
Little salty	15	17	11	16
Salty	14	11	16	18
Little seasoning	18	17	15	16
Seasoned	28	23	26	35
Firm texture	18	16	14	65
Tender texture*	54	54	58	28
Lamb flavor	25	18	25	26
Meat aroma*	47	48	41	60
Barbecue aroma	36	39	37	47
Pleasant aroma*	52	52	45	62
Residual fat	40	60	59	12
Residual flavor*	20	21	24	4
Tasty*	57	51	47	82
Crumbly*	34	41	41	4

Different letters (a–b) indicate significant difference between treatments (*P < 0.05).

^§^Evaluated in 9‐point hedonic scales varying from 1: disliked extremely to 9: liked extremely. Mixture 3: FAT 8.3%, CMC 1.7%, IN 0.0%; Mixture 4: FAT 5.0%; CMC 1.7%; IN 3.3%; Mixture 5: FAT 6.6%; CMC 1.7%; IN 1.7%; Mixture 8: FAT 5.9%; CMC 0.8%; IN 3.3%.

Cooked patties containing higher amount of CMC (mixtures 3, 4, and 5), which were similar based on instrumental techniques, were found to be crumblier, fattier, more moisty in their appearance, and with tender texture than mixture 8. This result is potentially attributed to the role of CMC on increasing moisture and fat retention on this type of product. In the study of Schuh et al. (2013), the addition of CMC caused a decrease on sausage firmness; samples contained 2% (w/w) CMC exhibited 486 N while the control batch 1,250 N. Gibis et al. (2015) reported that beef patties with tender texture had higher levels of CMC addition.

Considering that consumers often exhibit rather heterogeneous preference patterns, the segmentation of them into clusters characterized by similar preference patterns enables a more realistic view of the products performance. Consumer segmentation allows optimizing products for different segments, and identifying the drivers of liking (Berget, 2018). The acceptance data were segmented, and three groups of consumers with similar liking were identified. Table 4 presents the average acceptance for the identified segments. According to the results, segment 2 (with 19 consumers) did not like any of the patties, except the formulation M8. Consumers from the segment 1 (*n* = 42) liked moderately lamb patties M3, M4, and M5, and clearly preferred the formulation M8. On the other hand, participants from the segment 3 (*n* = 34) liked all of the patties and did not demonstrate any difference in terms of liking among the evaluated samples.

**Table 4:** Overall liking§ for the three segments of consumers of lamb patties

Segments of consumers			
	Segment 1 (*n* = 42)	Segment 2 (*n* = 19)	Segment 3 (*n* = 34)
M3	5.9^d^	4.5^e^	8.0^ab^
M4	6.2^cd^	3.6^e^	7.9^ab^
M5	5.8^d^	4.5^e^	7.8^ab^
M8	7.6^ab^	7.2^bc^	8.4^a^

§ Evaluated in 9‐point hedonic scales varying from 1: disliked extremely to 9: liked extremely.

Different letters (a–e) indicate significant difference between treatments (*p* < 0.05).

The caloric content of the selected mixtures ranged from 170.2 to 217.3 kcal/100 g (Table 5). The results of this study revealed that the replacement of FAT for different amounts of CMC and IN in the formulations of lamb patties affected the caloric value (*p* < 0.05), resulting in products less concentrated in energy source. Chizzolini, Zanardi, Dorigoni, and Ghidini (1999) reported that patties added with 20%–35% of fat have the caloric value range from 272 to 360 kcal/100 g. Products can be considered “reduced calorific value” options by presenting a reduction of caloric value >25% as compared to the original, as established in Resolution RDC No. 54/2012 for use of Complementary Nutrition Information (INC) in Food (Brazil, 2012). The present results indicated that lamb patties added of CMC and IN as fat replacers can be considered “reduced calorific value” products.

**Table 5:** Caloric value of lamb patties made with different levels of FAT, CMC, and IN

Samples	Calories (kcal/100 g)
Mixture 3	217.3^a^
Mixture 4	170.2^d^
Mixture 5	201.9^b^
Mixture 8	191.1^c^

Different letters (a–d) indicate significant difference between treatments (*p* < 0.05).

Mixture 3: FAT 8.3%, CMC 1.7%, IN 0.0%; Mixture 4: FAT 5.0%; CMC 1.7%; IN 3.3%; Mixture 5: FAT 6.6%; CMC 1.7%; IN 1.7%; Mixture 8: FAT 5.9%; CMC 0.8%; IN 3.3%.

## 4 CONCLUSION

The mixture M8 with 5.8% FAT, 0.8% CMC, and 3.3% IN can be effectively used to produce lamb patties with reduced fat while enhancing cooking yield and diameter reduction with no negative effect on texture profile and color parameters. The final product was highly accepted by consumers. Furthermore, based on the check‐all‐that‐apply results, it is not recommended the utilization of CMC level higher than 1% (w/w) due to the increase of undesirable sensory attributes as “residual flavor” and “crumbly.” Also, IN when added to lamb patties without CMC promoted a negative effect on cooking yield, diameter reduction, and texture profile.

## CONFLICT OF INTEREST

The authors notify that there are no conflicts of interest.

## AUTHOR CONTRIBUTIONS

Juliana M. Guedes‐Oliveira: Collected data, interpreted the results and drafted the manuscript. Bruno R. C. Costa‐Lima: Designed the study and reviewed the manuscript. Denize Oliveira: Designed sensory evaluation and interpreted the results, reviewed the manuscript. Adelino Cunha Neto: Collected data and reviewed the manuscript. Rosires Deliza: Designed sensory evaluation and interpreted the results, reviewed the manuscript. Carlos A. Conte‐Junior: Designed the study and reviewed the manuscript. Carlos Frederico Marques Guimarães: Collected data and reviewed the manuscript.

## ETHICAL STATEMENT

This study does not involve any human or animal testing.
